# Giant multiple caloric effects in charge transition ferrimagnet

**DOI:** 10.1038/s41598-021-91888-8

**Published:** 2021-06-21

**Authors:** Yoshihisa Kosugi, Masato Goto, Zhenhong Tan, Daisuke Kan, Masahiko Isobe, Kenji Yoshii, Masaichiro Mizumaki, Asaya Fujita, Hidenori Takagi, Yuichi Shimakawa

**Affiliations:** 1 Institute for Chemical Research, Kyoto University, Uji, Kyoto 611-0011 Japan; 2 Max Planck Institute for Solid State Research, 70569 Stuttgart, Germany; 3 Japan Atomic Energy Agency, Sayo, Hyogo 679-5148 Japan; 4 Japan Synchrotron Radiation Research Institute, Sayo, Hyogo 679-5198 Japan; 5 Magnetic Powder Metallurgy Research Center, AIST Chubu, Nagoya, Aichi 463-8560 Japan

**Keywords:** Magnetic properties and materials, Magnetic materials, Solid-state chemistry, Materials for energy and catalysis

## Abstract

Caloric effects of solids can provide us with innovative refrigeration systems more efficient and environment-friendly than the widely-used conventional vapor-compression cooling systems. Exploring novel caloric materials is challenging but critically important in developing future technologies. Here we discovered that the quadruple perovskite structure ferrimagnet BiCu_3_Cr_4_O_12_ shows large multiple caloric effects at the first-order charge transition occurring around 190 K. Large latent heat and the corresponding isothermal entropy change, 28.2 J K^−1^ kg^−1^, can be utilized by applying both magnetic fields (a magnetocaloric effect) and pressure (a barocaloric effect). Adiabatic temperature changes reach 3.9 K for the 50 kOe magnetic field and 4.8 K for the 4.9 kbar pressure, and thus highly efficient thermal controls are achieved in multiple ways.

## Introduction

Refrigeration and air conditioning account for a large amount of the world’s energy consumption, and effective thermal management is one of the critical issues that need to be resolved if we are to achieve the United Nations Sustainable Development Goals (SDGs)^1–3^. Caloric effects of solids can provide highly efficient energy conversion without using any hazardous gases and realize innovative and environmentally friendly energy systems^4–9^. In the caloric effects significant entropy changes in response to external fields lead to effective thermal conversions, and magnetocaloric, electrocaloric, and barocaloric effects are typical ones induced respectively by magnetic fields, electric fields, and pressure.

Magnetocaloric effects (MCEs) have been studied extensively in recent years, and an active magnetic regenerator system working at room temperature was actually developed^4,5,10–12^. Large MCEs at room temperature were reported in some magnetic intermetallics and alloys such as Gd_5_Si_2_Ge_2_, FeRh, and La(Fe,Si)_13_^13–15^. MCEs at low temperatures also attract attention for cryogen applications like for liquefying natural gases^16,17^. Not only alloys but also magnetic oxides including rare earth elements with large magnetic moments were also studied as MCE materials for hydrogen liquefying, which is expected to be green fuel instead of fossil fuel^18–20^. Electrocaloric effects (ECEs), on the other hand, were found in ferroelectric and pyroelectric compounds^21,22^. A practical temperature change through the ECE of the multilayer capacitor of PbSc_0.5_Ta_0.5_O_3_ was recently demonstrated^23^. Besides, not only the ferroelectrics but also VO_2_ was found to show the ECE^24^.

Some of the materials showing MCEs or ECEs also show barocaloric effect (BCE)^25–27^. Because a magnetic transition in a magnetic material having a magneto-striction property can be tuned by applying pressure, its caloric property can also be utilized by applying pressure. Similarly, a phase transition in a pyroelectric material is closely related to the material’s volume change, and thus the ECE can sometimes be induced by pressure. However, practical examples of such multiple caloric effects, where more than one type of caloric effects would arise in a single sample, are rarely reported. Because the caloric effects in such materials can be driven by different applied fiel﻿ds, effective thermal control can be achieved in multiple ways. Moreover, in a multicaloric effect, where the caloric effect is induced in multiple fields applied simultaneously, one can expand control of thermal properties with different order parameters. The exploration of novel multiple-caloric-effect and multicaloric materials, is therefore challenging but critically important for future technologies^7,8,28,29^.

In this paper we report that the *A*-site ordered quadruple perovskite structure ferrimagnetic oxide BiCu_3_Cr_4_O_12_ shows giant multiple caloric effects; that is, it shows both magnetocaloric and barocaloric effects and they are large. The compound exhibits large latent heat by the first-order charge transition at 190 K, and the corresponding giant entropy change can be utilized through the magnetocaloric and barocaloric effects respectively by applying magnetic fields and pressure. Electronic instability of a mixed-valence state of the constituent transition-metal cation and strong correlation in charge–spin–lattice degrees of freedom in BiCu_3_Cr_4_O_12_ are crucial for giving rise to the observed multiple caloric effects.

## Results and discussion

**Figure 1:**
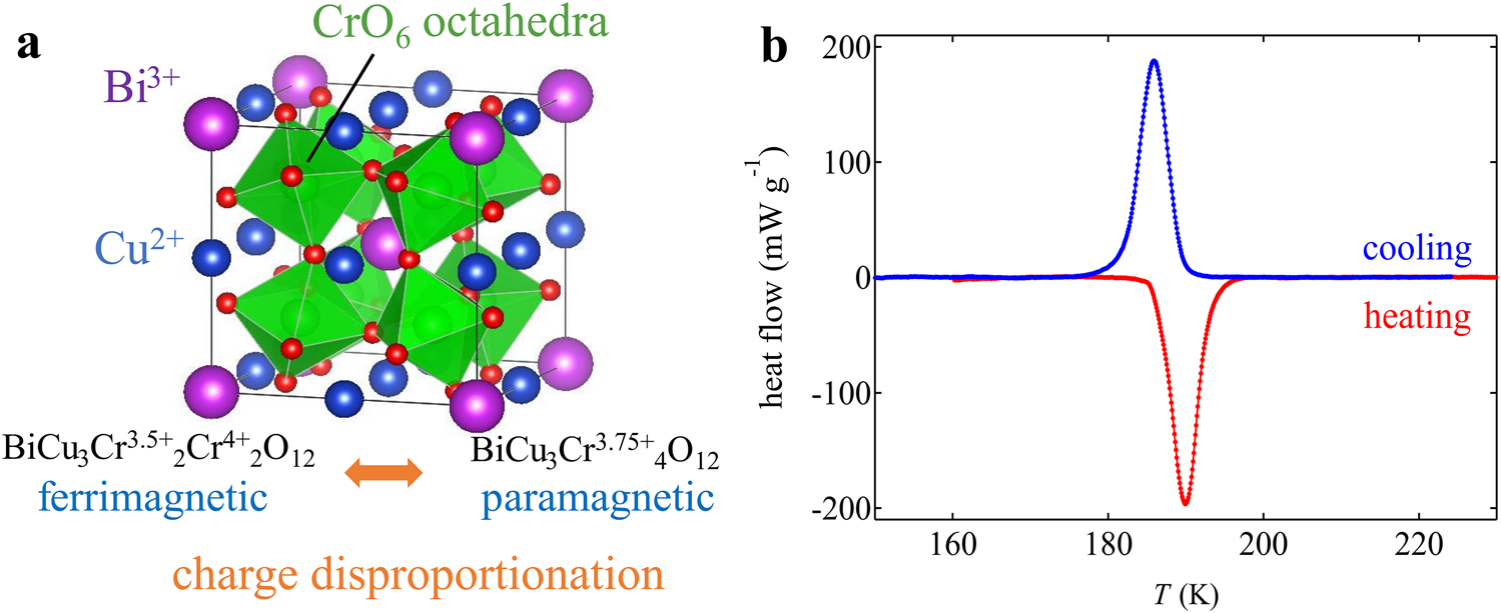
(**a**) Crystal structure of BiCu_3_Cr_4_O_12_. The compound shows charge disproportionation of Cr^3.75+^ at the *B* site to Cr^4+^ and Cr^3.5+^ at 190 K. The low-temperature Bi^3+^Cu^2+^_3_Cr^3.5+^_2_Cr^4+^_2_O_12_ phase shows ferrimagnetism by the antiferromagnetic coupling of *A*-site Cu and *B*-site Cr spins. (**b**) DSC curves of BiCu_3_Cr_4_O_12_ measured in cooling (blue) and heating (red) process.

BiCu_3_Cr_4_O_12_ crystallizes in the *A*-site ordered quadruple perovskite structure, where the Bi and Cu ions are 1:3 ordered at the *A* site and the Cr ions are located in the center of the corner-sharing *B*O_6_ octahedra in the *AB*O_3_ perovskite structure^30,31^. The compound contains mixed valence Cr^3.75+^ at room temperature and shows charge disproportionation of Cr^3.75+^ to Cr^3.5+^ and Cr^4+^ at 190 K to relieve the electronic instability. As a result, the high-temperature Bi^3+^Cu^2+^_3_Cr^3.75+^_4_O_12_ phase changes to the low-temperature Bi^3+^Cu^2+^_3_Cr^3.5+^_2_Cr^4+^_2_O_12_ phase, as reported previously (Fig. 1a)^32^. Details of the structure characterization and the phase transition changes are given in the Supporting information (1). This charge transition behavior is in sharp contrast to the intersite charge transfer seen in LaCu_3_Fe_4_O_12_ and BiCu_3_Fe_4_O_12_^33–35^. The asymmetric displacements of Bi with lone-pair electrons stabilize the order of the charge disproportionated Cr ions^32^. Importantly, this charge transition is a first-order transition and produces significant latent heat. As shown in the result of differential scanning calorimetry (DSC) measurement (Fig. 1b), the observed heat flow shows thermal hysteresis of 4 K between the cooling and heating process, and the latent heat estimated from the data on cooling is 5.23 kJ kg^−1^ (Table S2). The corresponding entropy change is 28.2 J K^−1^ kg^−1^, which is quite large for an oxide material.

**Figure 2:**
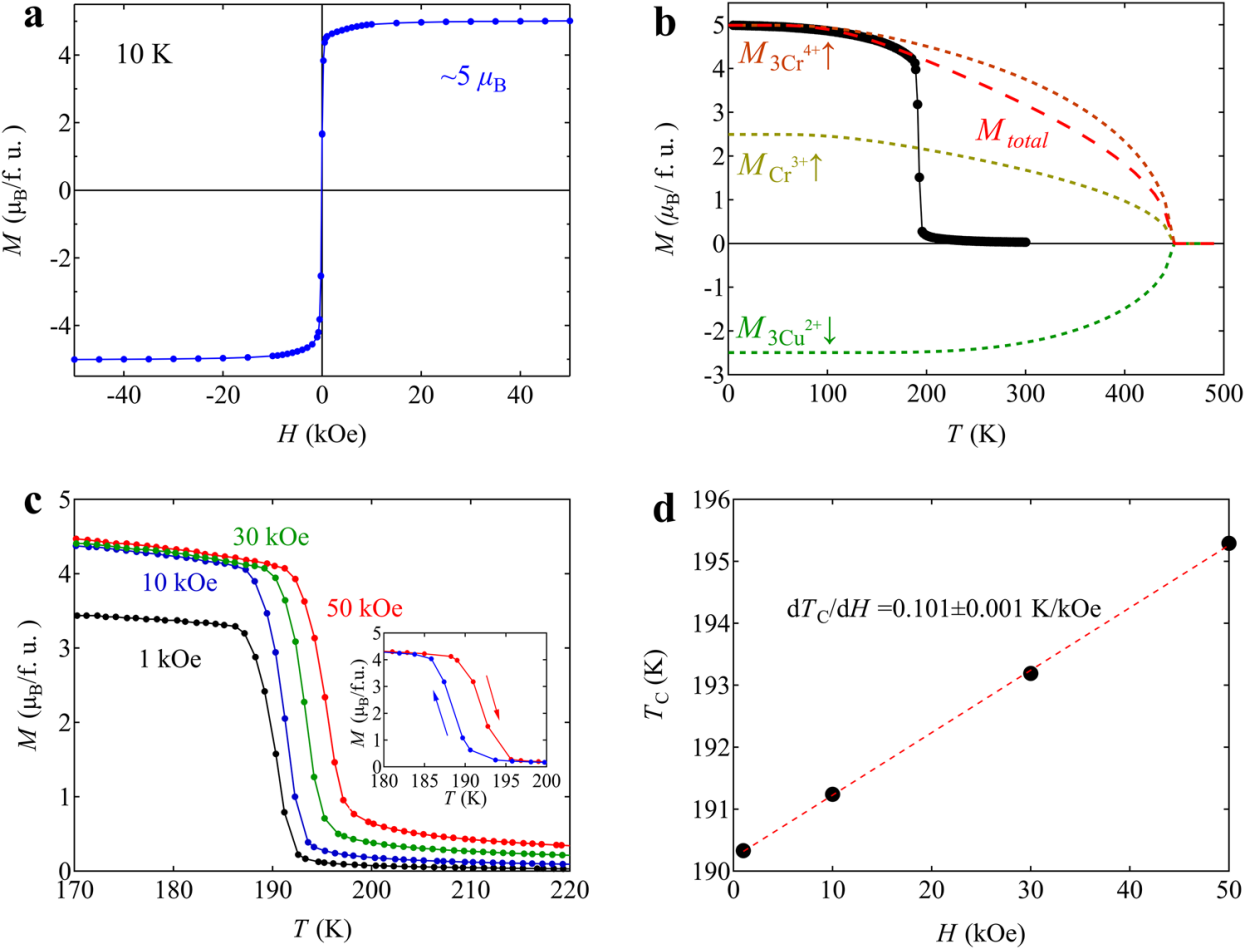
(**a**) Magnetization curve of the ferrimagnetic BiCu_3_Cr_4_O_12_ at 10 K. (**b**) Temperature dependence of magnetization for BiCu_3_Cr_4_O_12_ measured under 10 kOe. The red dash line represents the fitting for the total magnetization curve with the Brillouin function below the magnetic transition temperature. Contributions of Cr^3+^ (*S* = 3/2) ↑, 3Cr^4+^ (*S* = 1) ↑, and 3Cu^2+^ (*S* = 1/2) ↓ moments to the ferrimagnetization are shown in yellow, orange, and green dotted lines, respectively. (**c**) Temperature dependent magnetization curves for BiCu_3_Cr_4_O_12_ measured at various applied fields from 1 to 50 kOe in a field-cooling mode. The inset shows the thermal hysteresis between cooling (blue) and heating (red) at 10 kOe. (**d**) The magnetic field dependence of the transition temperature determined by the inflection points of magnetization curves. The dashed red line shows the linear fit of the data.

Note also that BiCu_3_Cr_4_O_12_ shows a ferrimagnetic transition accompanying the charge transition and that large ferromagnetic-like (ferrimagnetic) magnetization of about 5 μ_B_ is observed below the charge disproportionation transition temperature of 190 K. The observed magnetization is close to the value reported previously (5.65 μ_B_) but slightly smaller than that expected (6 μ_B_) for BiCu_3_Cr_4_O_12_ with ferrimagnetically ordered 3Cu^2+^, 1Cr^3+^, and 3Cr^4+^ spins (Fig. 2a)^32^. The behavior of this magnetic transition is also that of a first-order transition with the thermal hysteresis of about 3.5 K (inset of Fig. 2c) and far from that of a typical second-order transition driven by the superexchange magnetic interactions^36^. Given a simple ferrimagnetic structure consisting of the antiferromagnetically coupled *A*-site Cu (Cu^2+^ with *S* = 1/2) and *B*-site Cr (Cr^3+^ with *S* = 3/2 and Cr^4+^ with *S* = 1) spins, a fit to the temperature-dependent magnetization data with a Brillouin function (see details in the Supporting information (3)) gives an extrapolated magnetic transition temperature of about 450 K, which is much higher than (more than double) the actual magnetic transition temperature of 190 K (Fig. 2b). The magnetic entropy, which intrinsically has to be gradually changed at temperatures below 450 K, is thus abruptly yielded by the very sharp first-order magnetic (= charge) transition. Therefore, the observed large latent heat of BiCu_3_Cr_4_O_12_ should be related to the magnetic entropy change, as the large latent heat of the analogue compound NdCu_3_Fe_4_O_12_ is related to the magnetic entropy change of that compound^37^. Interestingly, the magnetization curves measured under various magnetic fields keep the sharp transition behaviors, but the transition temperature linearly increases with increasing the field (Fig. 2c). The linear field coefficient for the change of ferromagnetic transition temperature is 0.101 ± 0.001 K kOe^−1^ (Fig. 2d). Thus, importantly, the observed large latent heat, which is related to the magnetic entropy change, can be utilized through an MCE.

**Figure 3:**
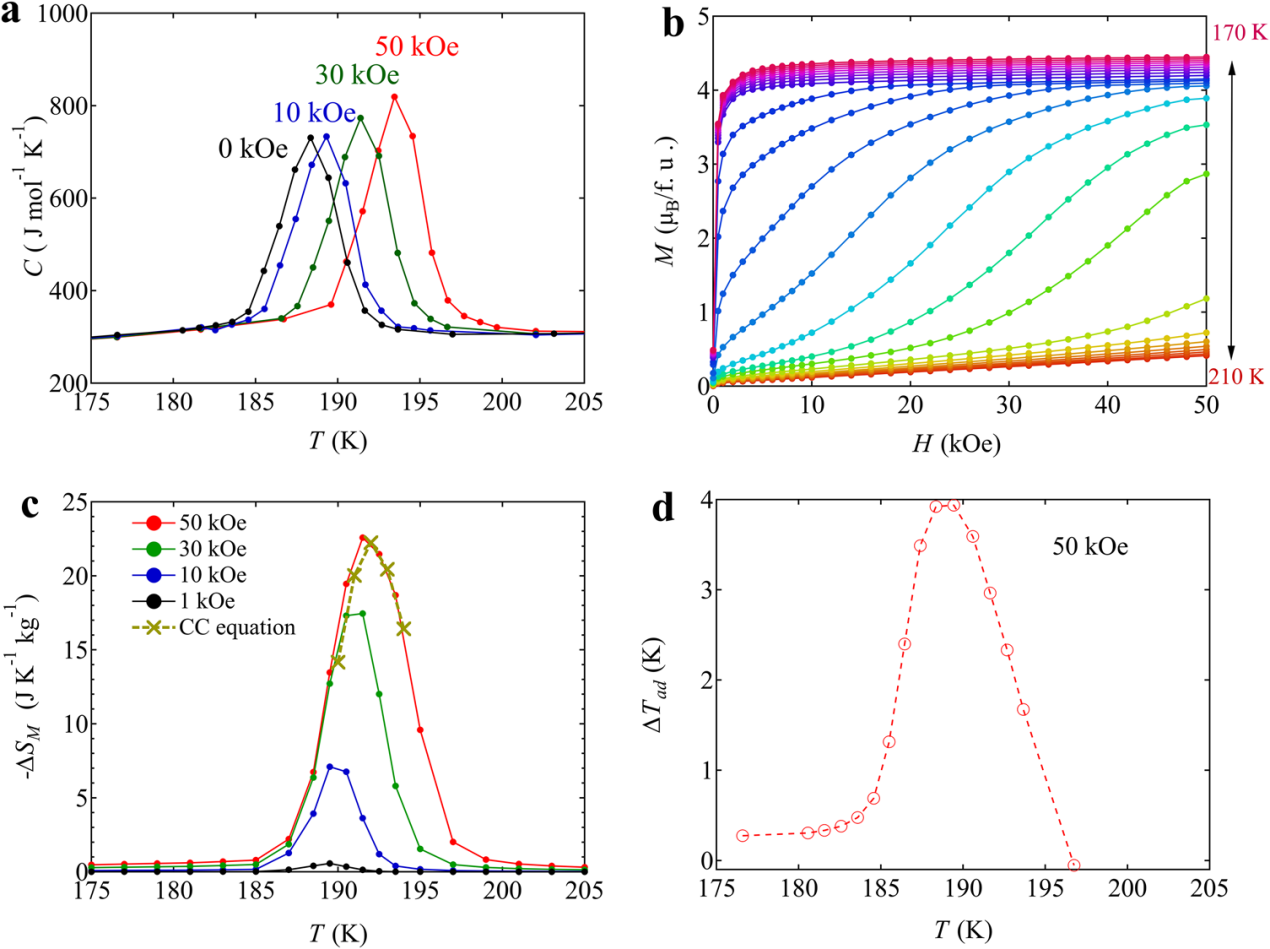
(**a**) Specific heat-capacity curves of BiCu_3_Cr_4_O_12_ measured under applied fields from 0 to 50 kOe. (**b**) Isothermal magnetization as a function of applied field for BiCu_3_Cr_4_O_12_ between 170 and 210 K. (**c**) Isothermal entropy changes for BiCu_3_Cr_4_O_12_ obtained from the magnetization changes measured under magnetic fields from 0 to 50 kOe. Yellow cross marks with a line represents the entropy changes estimated by the Clausius–Clapeyron equation using the magnetization data. (**d**) Calculated adiabatic temperature change induced by applying a magnetic field of 50 kOe.

Consistent with the increase in ferrimagnetic transition temperature under magnetic fields, specific heat-capacity curve measured under magnetic fields also shifts to a higher temperature with the linear field coefficient of 0.102 ± 0.001 K kOe^−1^, which is consistent with the value obtained in the magnetization measurement (Fig. 3a and Figure S2 in the Supporting information). At 50 kOe the specific heat capacity peak shifts by about 5 K and shows little overlap (16.5%) with that at 0 kOe. The result demonstrates that 83.5% of the whole latent heat can be utilized through the MCE at 50 kOe. Because the obtained specific heat capacity measured with a relaxation method by PPMS often underestimates the actual latent heat for a first-order transition^38^, we then evaluated the magnetic entropy change, $\Delta {S}_{M}$, from isothermal magnetizations as a function of applied magnetic fields. From the Maxwell relation

1 $${\left(\frac{\partial {S}_{M}}{\partial H}\right)}_{T}={\mu }_{0}{\left(\frac{\partial M}{\partial T}\right)}_{H},$$

the magnetic entropy is described as

2 $$\Delta {S}_{M}={\mu }_{0}{\int }_{0}^{H}{\left(\frac{\partial M}{\partial T}\right)}_{H}\mathrm{d}H.$$

With the isothermal magnetization measurement results from 170 to 210 K presented in Fig. 3b, the magnetic entropy change $\Delta {S}_{M}$ is thus estimated by the following formula:

3 $$\Delta {S}_{M}\left(\frac{2T+\Delta T}{2}\right)={\mu }_{0}{\int }_{0}^{H}\left\{\frac{M\left(T+\Delta T\right)-M\left(T\right)}{\Delta T}\right\}\mathrm{d}H,$$

where $\Delta T$ is the difference in temperature between which the isothermal magnetization data are taken (1 or 2 K in the present experiments) (see Fig. 3b,c). The resultant $\Delta {S}_{M}$ as a function of temperature is displayed in Fig. 3c. The maximum magnetic entropy change under 50 kOe reaches 22.6 J K^−1^ kg^−1^. Given that 83.5% of the transition entropy is released by the MCE at 50 kOe as mentioned above, the whole entropy change through the MCE should reach 27.1 J K^−1^ kg^−1^, which is comparable to that observed in DSC at zero field. This implies that almost all the latent heat originates from the magnetic entropy change and this large latent heat can be utilized through the MCE.

The magnetic entropy change can also be evaluated from the Clausius–Clapeyron equation:

4 $$\Delta {S}_{M}=-\Delta M{\left(\frac{\Delta {T}_{C}}{\Delta H}\right)}^{-1},$$

where $\Delta M$ is the difference between the magnetizations of the two phases and $\Delta {T}_{C}/\Delta H$ is the magnetic field dependence of the transition temperature^39,40^. The obtained $\Delta {S}_{M}$ at 50 kOe with $\Delta {T}_{C}/\Delta H$ determined by the inflection point in the magnetization curves (Fig. 2d) is also shown in Fig. 3c. It is noted that the magnetic entropy changes obtained from an indirect method with Maxwell relation are completely consistent with the results obtained from the Clausius–Clapeyron equation. The result is also consistent with the large latent heat observed in the present compound being primarily derived from the spin degree of freedom. The refrigerant capacity (RC), which is obtained by integrating $\Delta {S}_{M}$, is 101.1 J kg^−1^ at 50 kOe. The adiabatic temperature change caused by the MCE at 50 kOe,

5 $${\Delta T}_{ad}\left(T\right)={\lceil{T\left(S\right)}_{50 \,\mathrm{kOe}}-{T\left(S\right)}_{0 \,\mathrm{kOe}}\rceil}_{S}$$

is shown in Fig. 3d and the maximum adiabatic temperature change reaches 3.9 K at 189 K. Although magnetic hysteresis in a cycle of magnetic field change is not significant in the present compound (Figure S3a in the Supporting information), it causes hysteresis loss near the phase transition temperature^41,42^. The loss is estimated from the area of field magnetization loop as shown in Figure S3b in the Supporting information, and the maximum value is about 7 J kg^−1^ at 191 K and 50 kOe.

**Figure 4:**
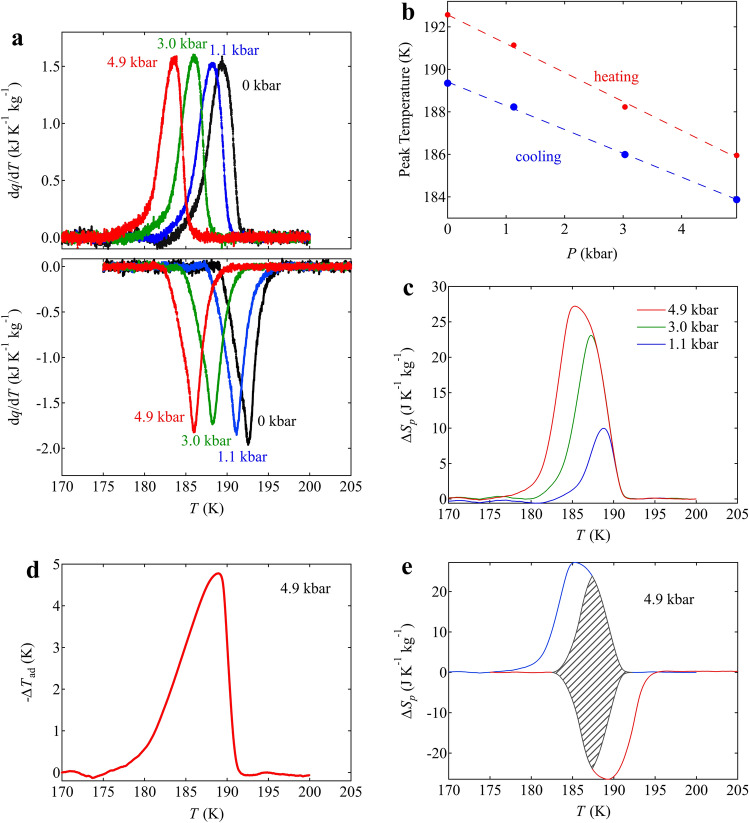
(**a**) Heat flow curves divided by cooling rates in BiCu_3_Cr_4_O_12_ measured under applied pressure from 0 to 4.9 kbar. Heat flow curves on heating are also shown in the lower panel. (**b**) Pressure dependences of peak temperature of the heat flow. The dashed lines show the linear fits of the data. (**c**) Corresponding entropies as a function of temperature under various applied pressures. Entropy is calculated using the equation described in the method section. The entropies on cooling are relative to the value at 200 K. (**d**) Calculated adiabatic temperature change induced by applying pressure of 4.9 kbar. (**e**) Isothermal entropy changes in both cooling and heating at 4.9 kbar. The shaded area represents a region of reversible entropy change.

Importantly, the large latent heat produced by the first-order charge disproportionation transition in BiCu_3_Cr_4_O_12_ can also be utilized through a BCE by applying hydrostatic pressure, as in the case of charge-transferred NdCu_3_Fe_4_O_12_^37^. Figure 4a shows the calorimetric curves obtained by differential thermal analysis (DTA) measurements at various pressures. Note that the exothermic peak shifts to a lower temperature under a pressure with the pressure coefficient of − 1.12 ± 0.02 K kbar^−1^ and − 1.36 ± 0.10 K kbar^−1^ respectively in cooling and heating as shown in Fig. 4b. The isothermal entropy changes produced by applying pressure are estimated by the following equation,

6 $${\Delta S}_{p}=S\left(T,p\right)-S\left(T,0\right).$$

The results are shown in Fig. 4c, and the maximum entropy change is found to reach 27.2 J K^−1^ kg^−1^ at 4.9 kbar on cooling. The result clearly demonstrates that the large entropy change observed in the DSC measurement is utilized through the BCE at 4.9 kbar. The corresponding adiabatic temperature change at 4.9 kbar,

7 $${\Delta T}_{ad}\left(T\right)={\lceil{T\left(S\right)}_{4.9\,\mathrm{ kbar}}-{T\left(S\right)}_{\mathrm{ambient}}\rceil}_{S}$$

is seen in Fig. 4d, and the maximum value reached 4.8 K at 189 K. The RC value at 4.9 kbar on cooling is 140 kg^−1^. The reversible temperature range, which is determined from the DTA measurements on cooling and heating under pressure, is also shown in Fig. 4e. The BCE is reversible between 185 and 189 K, and a higher pressure than 4.9 kbar is necessary for utilizing the full entropy change in BCE by the phase transition.

**Figure 5:**
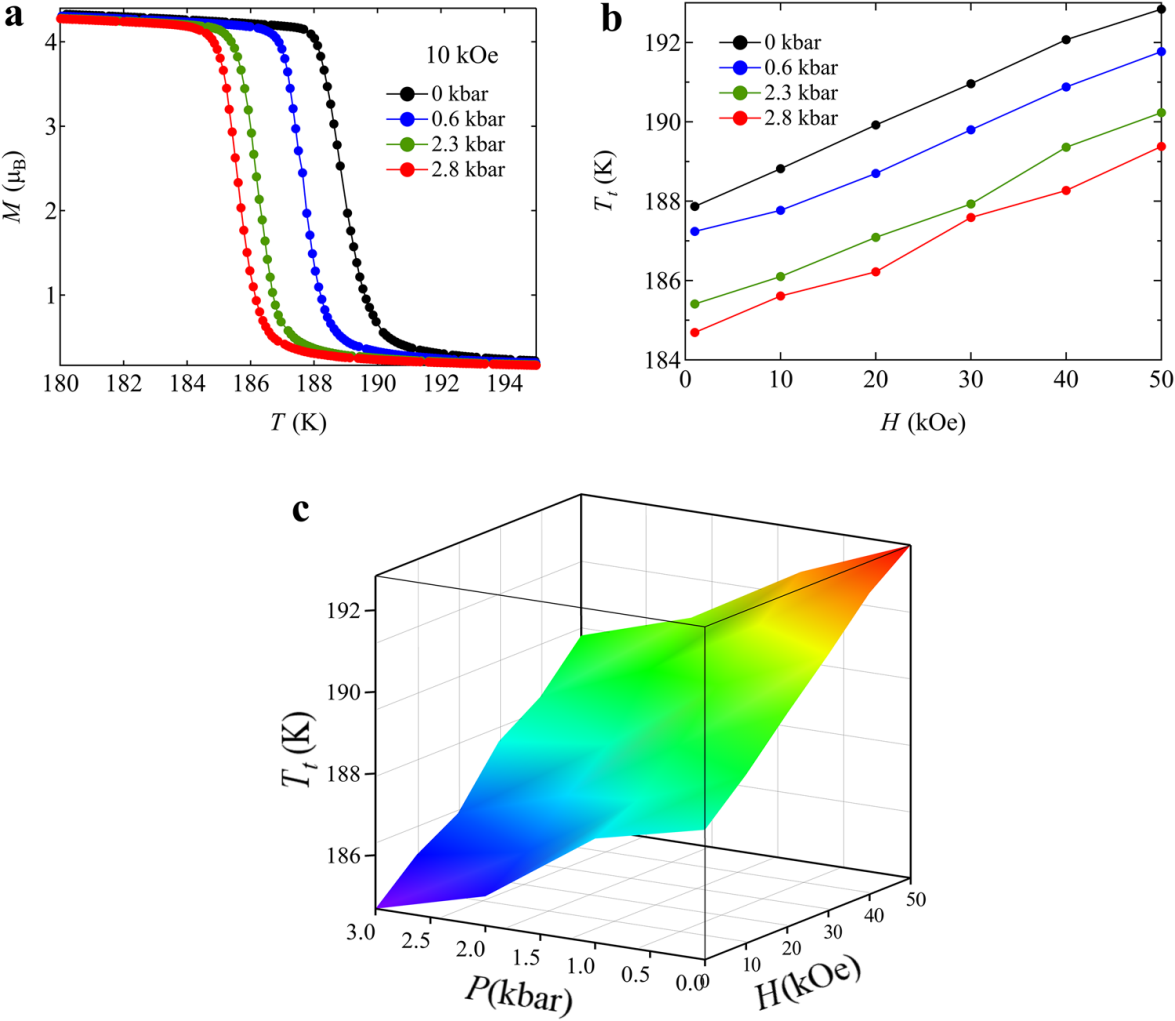
(**a**) Temperature dependent magnetization for BiCu_3_Cr_4_O_12_ measured under a 10 kOe magnetic field and various pressure. (**b**) Variation in phase transition temperature *T*_*t*_ by applying both magnetic fields and pressure. (**c**) 3D plot of phase transition temperature. The obtained planar surface represents the change of phase transition temperature by applying both magnetic fields and pressure.

The present BiCu_3_Cr_4_O_12_ exhibits both the MCE and the BCE. The large latent heat produced by the charge disproportionation transition can be utilized by applying both magnetic fields and hydrostatic pressure. An important point in the compound is that the phase transition can be tuned by applying fields in multiple ways while keeping the first-order sharp behaviors. Therefore, the large latent heat produced by the phase transition is also utilized in multiple ways. Although we presented the experimental results of MCE and BCE separately, we believe that the observed large entropy changes can be utilized by applying both magnetic fields and pressure simultaneously and thus the compound is multicaloric, because the charge, the spins, and the lattice are strongly coupled in the present BiCu_3_Cr_4_O_12_. Actually, as shown in Fig. 5, the phase transition temperature can be tuned by applying both magnetic fields and pressure simultaneously. As indicated by the linear field (magnetic field and pressure) dependences of the phase transition temperature, a planar surface separates the charge-uniform and charge-disproportionated phases (Fig. 5c). This multicaloric feature naturally provides us access to a wider range of control parameters, such that the phase transition where the entropy changes significantly can become accessible with fields in a broader window of temperature. An applied pressure to achieve a certain caloric effect, for example, can be tuned under a magnetic field. An effective magnetic field for utilizing the latent heat with the MCE can be controlled by applying pressure. In principle, it should be possible to eliminate the hysteresis as seen in the Fe_49_Ph_51_ alloy^43^.

The phase transition in BiCu_3_Cr_4_O_12_ is primarily caused by the charge instability of the high and mixed valence state of Cr^3.75+^, which induces the charge disproportionation transition^26^. An important point is that the magnetic states of constituent cations also change according to the change in the charge states. Each magnetic moment abruptly appears and simultaneously order themselves at the charge transition temperature, and the behaver is completely different from the order–disorder-type transition of the magnetic moments in most of the magnetic materials. This unusual first-order magnetic transition yields the large magnetic entropy change. The magnetic entropies are thus significantly changed by the charge transition. Importantly, charge, spin, and lattice degrees of freedom in BiCu_3_Cr_4_O_12_ are strongly coupled, and as a result, the charge transition is tuned by applying external fields like agnetic fields and pressure. Therefore, the large magnetic entropy change produced by the charge transition is controlled by different kinds of applied fields and can be utilized through the caloric effects. The charge transition in the charge–spin–lattice coupled system gives rise to the present large multiple caloric effects.

## Conclusions

We discovered that the quadruple perovskite structure oxide BiCu_3_Cr_4_O_12_ showed large multiple caloric effects; that is, it showed both magnetocaloric and barocaloric effects produced by the charge transition. The large latent heat of 5.23 kJ kg^−1^ and the corresponding entropy change of 28.2 J K^−1^ kg^−1^ are utilized by applying both magnetic fields and pressure. The multiple caloric effects are primarily driven by the magnetic entropy change in the unusual first-order magnetic transition accompanying the charge disproportionation transition of the mixed valence Cr^3.75+^. The charge, the spins, and the lattice are strongly correlated in the compound, and thus the phase transition caused by the electronic instability is tuned by multiple factors. The corresponding entropy changes are therefore able to be utilized through the multiple caloric effects. With this material, adiabatic temperature changes reach 3.9 K for the 50 kOe magnetic field and 5.4 K for the 4.9 kbar pressure, and thus highly efficient thermal controls are achieved in multiple ways. The present results demonstrate that a phase transition in charge–spin–lattice coupled system can be utilized as thermal control through multiple caloric effects. Our results open a new avenue to develop novel caloric materials.

## Methods

A polycrystalline BiCu_3_Cr_4_O_12_ sample was prepared by solid-state reaction under a high-pressure and high-temperature condition. The sample was synthesized from the stoichiometric mixture of Bi_2_O_3_, CuO, CrO_2_, and Cr_2_O_3_. The mixture was pressed at 9 GPa and heated at 1273 K for 30 min and then cooled to room temperature. The pressure was slowly released after the heat treatment. The obtained sample was confirmed to be a single phase by synchrotron X-ray diffraction (SXRD). The results of structure analysis are given in Supporting information. The structure parameters were refined by Rietveld analysis using the RIETAN-FP program^44^. The crystal structure figures were drawn using the VESTA software^45^.

The magnetization data of the powder samples were collected with a SQUID magnetometer (Quantum Design MPMS XL) between 10 and 300 K under magnetic fields from 1 to 50 kOe. Isothermal magnetization data were collected from 0 to 50 kOe between 170 and 210 K.

Differential scanning calorimetry (NETZSCH DSC3500) was carried out at heating and cooling rates of 10 K min^−1^. The heat flow curves were obtained by subtracting the base change. The latent heat $Q$ and the entropy change $\Delta S$ associated with the transition were calculated as ${{Q}}={\int }_{{{T}}_{{b}}}^{{{T}}_{{a}}}{\mathrm{d}}{\dot{Q}}/{\dot{T}}{\mathrm{d}}{T}$ and $\Delta {S}={\int }_{{{T}}_{{b}}}^{{{T}}_{{a}}}\left\{(-{\mathrm{d}}{\dot{Q}}/{\dot{T}})/{T}\right\}{\mathrm{d}}{T}$, where ${\mathrm{d}}{\dot{Q}}$ is the heat flow and $\dot{T}$ is the cooling or heating rate. The heat-capacity measurements under various magnetic fields were performed by a commercial calorimeter (Quantum Design PPMS) using the heat relaxation method in the heating process. A pellet sample was used and was fixed on the sample holder by Apiezon N grease. RC in MCE was estimated by integrating $\Delta {S}_{M}$ between the temperatures at the half maximum of the peak in $\Delta {S}_{M}$.

Differential thermal analysis (DTA) measurements were carried out by using a pressure cylinder made of Cu–Be. The details of the equipment and the setup are described in the reference^46^. A T-type thermocouple was adhered with varnish to each sample and the CuO reference pellet. The DTA cell with Daphne7373 pressure medium was inserted in the pressure cylinder. Hydrostatic pressure was applied by a hydraulic cylinder via a piston. The sample temperature was controlled using liquid nitrogen. The heat flow (divided by cooling rate) curves were obtained with the DTA signal *δT* as d*q*/d*T* =  − *AδT*$/\dot{T}$. Because the proportional constant *A* scarcely changed during measurements with the same setup conditions under pressure, the *A* value was determined from the heat flow $\dot{Q}$ measured by DSC under an ambient condition. The entropy was evaluated as $S(T,p)={\int }_{{T}_{b}}^{{T}_{a}}\left\{(-A\delta T(p)/\dot{T})/T\right\}\mathrm{d}T$. The isothermal entropy change ${\Delta S}_{p}$ at each pressure was calculated as ${\Delta S}_{p}=S\left(T,p\right)-S(T,0)$. The reversible temperature range was determined from the DTA measurements on cooling and heating under pressure. RC in BCE was estimated by integrating $\Delta S$ between the cold and hot reservoir temperatures, where $\Delta S$ was a half value of the maximum.

## Supplementary Information

Supplementary Information.
