# Supplementary material for: Giant multiple caloric effects in charge transition ferrimagnet
Source: Sci Rep. 2021 Jun 21;11:12682. doi: 10.1038/s41598-021-91888-8 (PMC8217487; doi:10.1038/s41598-021-91888-8)
Supplement: Supplementary file 1 — Supplementary Information. [file 41598_2021_91888_MOESM1_ESM.pdf]

Supporting information for

Giant multiple caloric effects in charge transition ferrimagnet

Yoshihisa Kosugi[1], Masato Goto[1], Zhenhong Tan[1], Daisuke Kan[1], Masahiko Isobe[2], Kenji Yoshii[3], Masaichiro Mizumaki[4], Asaya Fujita[5], Hidenori Takagi[2], and Yuichi Shimakawa[1,*]

[1] *Institute for Chemical Research, Kyoto University, Uji, Kyoto 611-0011, Japan*

[2] *Max Planck Institute for Solid State Research, D-70569 Stuttgart, Germany*

[3] *Japan Atomic Energy Agency, Sayo, Hyogo 679-5148, Japan*

[4] *Japan Synchrotron Radiation Research Institute, Sayo, Hyogo 679-5198, Japan*

[5] *Magnetic Powder Metallurgy Research Center, AIST Chubu, Nagoya, Aichi 463-8560, Japan*

This Supporting information consists of (1) crystal structure at 300 K and temperature-dependent structure analysis, (2) DSC measurement results, (3) ferrimagnetic magnetization analysis, (4) magnetic field dependence of peak temperature in heat capacity curves, and (5) isothermal magnetization curves and the hsiterisis loss in MCE of $BiCu_3Cr_4O_{12}$.

*e-mail: shimak@scl.kyoto-u.ac.jp

(1) Structure analysis with the SXRD data collected at 300 K confirm that $BiCu_3Cr_4O_{12}$ synthesized in the present study crystalizes in a quadruple perovskite structure with the space group $Im\bar{3}$. Temperature-dependent SXRD data also confirm the phase transition due to charge disproportionation transition. All the structure analysis results are essentially the same as those reported previously[1].

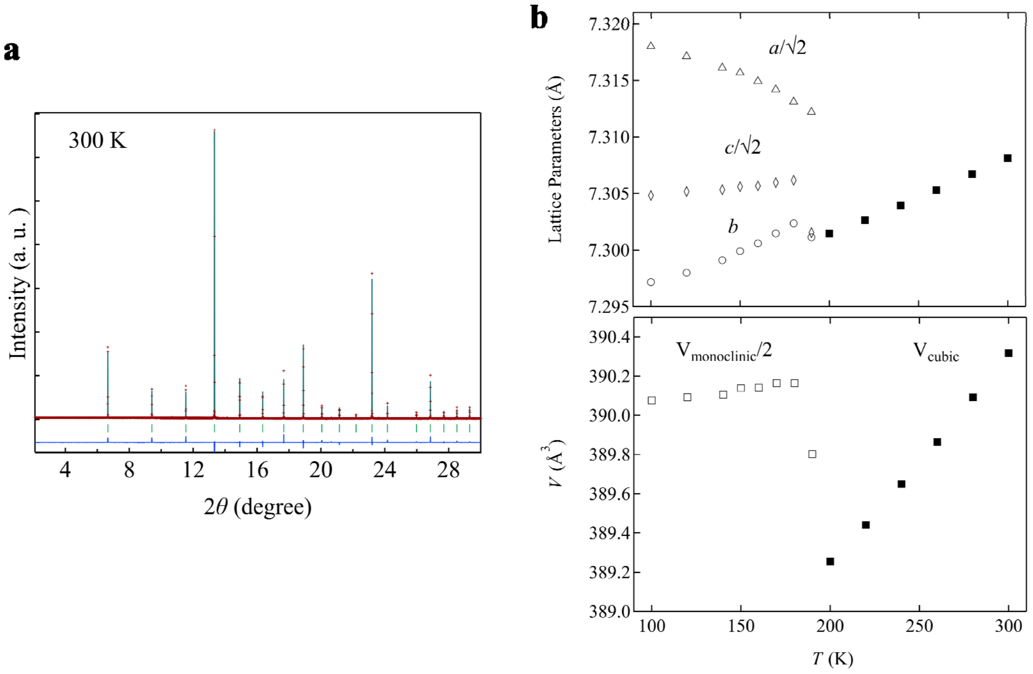

Figure S1 (**a**) The SXRD patterns and the results of Rietveld structure refinements for $BiCu_3Cr_4O_{12}$ at 300 K. The wavelength for the measurements is 0.49950 Å. Vertical bars indicate the Bragg reflection peak positions of $BiCu_3Cr_4O_{12}$. The bottom curves (blue) show the difference between observed and calculated intensities. (**b**)Temperature dependence of lattice parameters (upper part) and volume (lower part).

Table S1 Refined structure parameters of $BiCu_3Cr_4O_{12}$ at 300 K.

**atom**	**site**	***x***	***Y***	***z***	***B*** **(Å^2)**
Bi	2*a*	0	0	0	1.11(1)
Cu	6*b*	0	1/2	1/2	0.19(1)
Cr	8*c*	1/4	1/4	1/4	0.10(1)
O	24*g*	0	0.3010(3)	0.1787(3)	0.1

Lattice parameter: a = 7.30813(2) Å;Volume :V = 390.318(1) Å^3; R_{wp} = 5.676%

(2) Thermal properties for $BiCu_3Cr_4O_{12}$. The thermal hysteresis in DSC confirms the charge disproportionation transition is a first-order transition.

Table S2. Peak temperature, latent heat and the corresponding entropy change obtained by DSC measurements on heating and cooling.

	**Heating**	**Cooling**
Peak temperature (K)	189.9	185.9
Latent heat (kJ kg^{-1})	5.08	5.23
Transition entropy change (**J K^{-1} kg^{-1}**)	26.8	28.2

(3) Temperature-dependent magnetization data show that the transition is a first-order transition, and the intrinsic magnetic correlation temperature extrapolated from the data is suggested to be much higher than the observed transition temperature of 190 K.

Here a simple ferrimagnetic model is considered. In the mean-field theory, temperature dependence of magnetization is described by the following equations:

$$M(T) = M_S \mathrm{B}_J \left(\frac{J g_J \mu_B \lambda (M + H)}{k_\mathrm{B} T} \right)$$

and

$$\mathrm{B}_J(y) = \frac{2J+1}{2J} \coth\left(\frac{2J+1}{2J} y \right) - \frac{1}{2J} \coth\left(\frac{1}{2J} y \right),$$

where M_S, B_J, J, g_J, λ, and H respectively are saturation magnetization, Brillouin function, total angular moment, g-factor, mean field coefficient, and external magnetic field. In the present ferrimagnet $BiCu_3Cr_4O_{12}$, the antiferromagnetic interaction between the spin moments at the A and B sites is considered: $BiCu^{2+}(\downarrow;\ S = 1/2)_3Cr^{3+}(\uparrow;\ S = 3/2)Cr^{4+}(\uparrow;\ S = 1)_3O_{12}$. As reported previously,[1] the charge disproporationated state of $BiCu_3Cr_4O_{12}$ was described as $BiCu^{2+}{}_3Cr^{3.5+}{}_2Cr^{4+}{}_2O_{12}$, but the magnetic structure was considered as that described above. The randomly distributed Cr^{3+} and Cr^{4+} spins at the $Cr^{3.5+}$ site are ferromagnetically coupled. Therefore, the total ferrimagnetic moments should be $-3\times 1Cu^{2+}(1) + 1\times Cr^{3+}(3) + 3\times Cr^{4+}(2) = 6\ \mu_B$. Each magnetization of the spin sublattices of A-site Cu^{2+}, B-site Cr^{3+}, and B-site Cr^{4+} is given as follows:

$$M_{3\mathrm{Cu}^{2+}}(T) = M_{S,3\mathrm{Cu}^{2+}} \mathrm{B}_{1/2} \left(\frac{1}{2} \frac{g \mu_\mathrm{B} \lambda (M_{\mathrm{Cr}^{3+}} + M_{3\mathrm{Cr}^{4+}})}{k_\mathrm{B} T} \right)$$

$$M_{\mathrm{Cr}^{3+}}(T) = M_{S,\mathrm{Cr}^{3+}} \mathrm{B}_{3/2} \left(\frac{3}{2} \frac{g \mu_\mathrm{B} \lambda M_{3\mathrm{Cu}^{2+}}}{k_\mathrm{B} T} \right)$$

$$M_{3\mathrm{Cr}^{4+}}(T) = M_{S,3\mathrm{Cr}^{4+}} \mathrm{B}_{1} \left(\frac{g \mu_\mathrm{B} \lambda M_{3\mathrm{Cu}^{2+}}}{k_\mathrm{B} T} \right),$$

where $M_{S,3\mathrm{Cu}^{2+}}$, $M_{S,\mathrm{Cr}^{3+}}$, and $M_{S,3\mathrm{Cr}^{4+}}$ are the saturation magnetizations of each spin sublattice. The weight of each spin sublattice is considered. The observed magnetization data below the magnetic transition temperature is fitted to the total magnetization

$$M_{total}(T) = M_{3\mathrm{Cr}^{4+}}(T) + M_{\mathrm{Cr}^{3+}}(T) - M_{3\mathrm{Cu}^{2+}}(T)$$

by numerical calculations.

(4) The field dependence of peak temperature shifts 0.102 K kOe^{-1} is consistent with the value obtained from the magnetization curves (0.101 K kOe^{-1}).

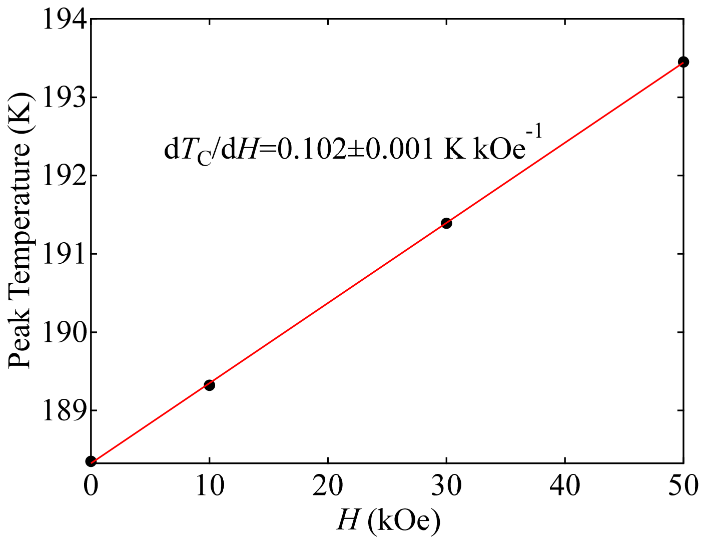

Figure S2 Field dependence of peak temperature for heat capacity curves. Red line shows the linear fit of the data.

(5) Although magnetic hysteresis in a cycle of magnetic field change is not significant in the present $BiCu_3Cr_4O_{12}$, it causes hysteresis loss near the phase transition temperature. The isothermal magnetization curves between 180 and 204 K are shown in Figure S3a in the Supporting information, and the loss is estimated from the area of field magnetization loop. The maximum value of the hysteresis loss in MCE is about 7 Jkg^{-1} at 191 K and 50 kOe.

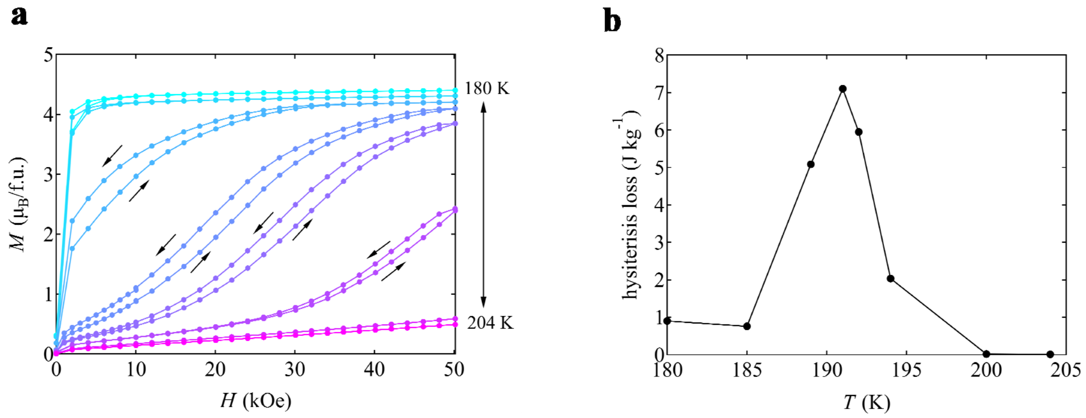

Figure S3. (**a**) Isothermal magnetization curves between 180 and 204 K measured during both magnetizing and demagnetizing processes. (**b**) Hysteresis loss in MCE of $BiCu_3Cr_4O_{12}$.

Reference

1. Etter, M. *et al.* Charge disproportionation of mixed-valent Cr triggered by Bi lone-pair effect in the *A*-site-ordered perovskite $BiCu_3Cr_4O_{12}$. *Phys. Rev. B* **97**, 195111 (2018).
